# Tracking Study on the Relapse and Aftercare Effect of Drug Patients Released From a Compulsory Isolated Detoxification Center

**DOI:** 10.3389/fpsyt.2021.699074

**Published:** 2022-01-17

**Authors:** Nian Liu, Zekai Lu, Ying Xie

**Affiliations:** Department of Sociology, School of Public Administration, Guangzhou University, Guangzhou, China

**Keywords:** compulsory detoxification, drug abstainers, relapse rate, experimental design, survival time, Bayesian estimation, logistic regression

## Abstract

**Background and Aims:**

There are no accurate statistical data on the relapse rate of drug abstainers after compulsory detoxification in China. This study aimed to collect relapse data for drug abstainers through follow-up visits, verify the effectiveness of professional social worker services and explore significant factors affecting relapse.

**Design and Setting:**

The drug abstainers released from Guangzhou T Compulsory Isolated Detoxification Center were randomly divided into two groups. The difference between the experimental group and the control group is that assistance services were provided by social workers to the former.

**Participants:**

The study included 510 drug abstainers released from T Center, including 153 in the experimental group and 357 in the control group.

**Measurements:**

Demographic information, history of drug abuse, and motivation for drug rehabilitation (SOCRATES) were collected 1 month prior to drug abstainer release from compulsory detoxification. Then, the relapse situation after their release was tracked according to fixed time points.

**Findings:**

The overall relapse rate of 510 drug abstainers after their release from compulsory detoxification was 47.6%. The average survival time to relapse based on survival analysis was 220 days (*N* = 486), as calculated with Bayesian estimation by the MCMC method. The average survival times to relapse of the experimental group and control group were 393 and 175 days, respectively. By taking the specific survival time as the dependent variable and the group as the control variable (*OR* = 25.362), logistic regression analysis showed that marital status (*OR* = 2.666), previous compulsory detoxification experience (*OR* = 2.329) and location of household registration (*OR* = 1.557) had a significant impact on the survival time to relapse.

**Conclusions:**

The occurrence of relapse among drug patients released from compulsory detoxification can be delayed effectively through the intervention of professional social worker services. Regardless of whether patients receive aftercare after compulsory detoxification, drug-using patients who are single, have multiple detoxification experiences and whose households are registered in other provinces deserve special attention. Relevant suggestions to avoid relapse are provided.

## Introduction

Drugs are a public nuisance in society. Drug abuse has always been a major social issue that has seriously affected China's social stability and economic development and individuals' livelihoods. With the continued proliferation of global drug problems, especially the transition from traditional drugs to new drugs, the domestic drug situation in China remains severe and complex (1). According to the *China Drug Situation Report 2016* (2), as of the end of 2016, there were 2.505 million drug abusers in China. In that same year, China arrested 1.006 million drug abusers, including 445,000 registered newly discovered drug abusers, 357,000 undergoing compulsory isolated detoxification by law, 245,000 ordered to undergo community-based detoxification and 59,000 ordered to undergo community-based rehabilitation. By the end of 2020, there were still 1.801 million drug abusers nationwide, and 427,000 drug users were investigated and dealt with throughout the year, of which 155,000 were newly discovered drug abusers, 149,500 were subjected to compulsory isolated detoxification, and 99,000 were ordered to undergo community-based detoxification (3). Guangdong Province suffers the most serious drug abuse problem in China. In recent years, drug problems have become increasingly serious there. In 2014, the number of registered drug abusers in Guangdong Province was 470,000, and it reached 582,000 by the end of 2015, an increase of 100,000 in 1 year. Compared with the national data from the same period, the number of drug abusers in Guangdong is the highest in the country (4), accounting for approximately one quarter of the national total and causing a loss of more than RMB 100 billion to society every year. With the launch of a new round of a “severe crackdown on crimes”^1^ across the country, the number of registered drug users in Guangdong fell to 330,000 in 2019, but it is still the highest in the country, and the base of drug users is still very large (5).

Drug addiction in the medical field is considered to be cyclical chronic intoxication caused by repeated drug use and is a serious intractable, recurrent brain disease that is prone to relapse (6). The pathological changes of drug addiction are caused by brain atrophy and the gradual mutation of brain cells by drugs (7). The physiological changes after drug addiction are that the drug inhibits the secretion function of the endogenous morphine produced in the brain, leading to physiological and psychological dependence on the drug, causing the body to lose its self-balance and self-control ability and resulting in the dysfunction of various systems (8). Because of the brain lesions associated with drug use, drug patients are very vulnerable to relapse after receiving drug treatment.

Reflecting the pathological characteristics of drug abuse, the relapse rate of Chinese drug abstainers has remained high. The 2016–2018 report on China's drug situation showed that the numbers of relapsers arrested in the 3 years were 600,000, 532,000, and 504,000, respectively. The number of relapsers has always accounted for more than half of the number of drug users arrested in a given year. According to public reports, the relapse rate in many areas of China is as high as 90% (9). Determining how to prevent relapse and effectively reduce the relapse rate of drug abstainers is a worldwide challenge (10). Zheng and Fang (11) conducted a follow-up visit with 443 heroin addicts in their first week after discharge following hospitalization for voluntary detoxification and found that the experience of detoxification greatly affects the relapse rate. The relapse rate of those who experienced detoxification once was 59.91%, and the relapse rate of those who experienced multiple detoxifications was as high as 88%. A Spanish follow-up study of 108 drug patients who received detoxification treatment in the hospital showed that the relapse rate within 6 months after discharge was 72.2% (12), and opioid-dependent patients were most likely to relapse. Ivers (13) conducted a follow-up study of 143 patients who received 14 months of detoxification treatment in Ireland and found that within 9 months, the abstinence rate of patients without formal aftercare was only 6%, and the abstinence rates of the outpatient and inpatient aftercare groups were 50 and 67%, respectively. The overall abstinence rate of 143 patients was 50%, and whether there were corresponding aftercare services after ending drug addiction was an important factor affecting the relapse rate. Relapse is the greatest obstacle to the rehabilitation of drug abstainers.

Although medical treatment has a very important role in detoxification, drug rehabilitation cannot rely solely on medical abstinence, and it involves factors such as neurobiology, psychiatry, and sociology (14). Drug abstainers try to maintain drug rehabilitation, but due to internal factors such as motivation for addiction treatment and self-concept (15) or external factors such as drug-related stimulation, family environment and interpersonal loneliness (16), it is impossible for them to entirely free themselves from entanglement with drugs. Relapse usually occurs within 1 year after recovery from physiological drug addiction (17). Once drug abstainers come into contact with drugs again, previous efforts toward drug rehabilitation will be wasted. Domestic and foreign studies have noted that training and enhancing the motivation for drug rehabilitation of drug abstainers and teaching and guiding drug abstainers to master the skills needed to cope with high-risk relapse situations has a significant impact on reducing the relapse rate (16, 18). On the one hand, anti-drug abuse work should make efforts to prevent increases in new drug abusers, cut off the supply of drugs at the source, and strengthen anti-drug publicity and education. On the other hand, for drug abstainers who have already used drugs, it is also very important and arduous to carry out scientific drug detoxification to cure their behavioral and mental compulsion related to chronic drug addiction-related tendencies (8, 19) and reduce their relapse rate. Moreover, after detoxification treatment, it is difficult for drug abstainers to eliminate the effects of drugs on their psychology, physiology and social life, and they find it difficult to cope with high-risk situations involving drugs and achieve true “detoxification,” resulting in reuse of drugs (20).

At present, compulsory isolation for drug rehabilitation is still the leading detoxification measure for drug users under detoxification, and there are no accurate statistical data on the relapse rate of drug abusers after compulsory isolated detoxification in China. According to the *Anti-drug Law of the People's Republic of China* (21), drug abusers under compulsory isolated detoxification experience failure in community-based detoxification. If a drug abuser is detected by the public security organ for the first time, the public security organ will generally order the drug abuser to undergo 3-year community-based detoxification in the place where his or her registered permanent residence or fixed residence is located. If the drug abuser is severely addicted and he or she is assessed by a professional physician as “unlikely to quit drug addiction through community-based detoxification,” he or she can be directly subjected to compulsory isolated detoxification. When a drug abuser under community-based detoxification is in one of the following circumstances, the public security organ will impose compulsory isolated detoxification for drug rehabilitation: ①refusing to accept treatment with community-based detoxification; ②ingesting or injecting drugs during the period of treatment with community-based detoxification; ③seriously violating the agreement on treatment in community-based detoxification (such as failure to perform a urine test or hair test regularly or long-term loss of contact); ④relapsing into ingesting or injecting drugs after community-based detoxification or after compulsory isolated detoxification (21).

Due to the lack of relapse rate data, the corresponding relapse prevention methods and effectiveness evaluations lack evidence and precise guidance. Previous studies on relapse rates have mainly focused on cross-sectional studies (22), and longitudinal tracking data are rare in China. In addition to medical treatment, in recent years, the Chinese government has begun to promote the development of aftercare services for drug patients to address relapse more effectively. How effective are these aftercare services? This is also a question that needs to be answered. Drug rehabilitation measures can be scientifically formulated, and the effectiveness of existing drug rehabilitation efforts can be improved only by obtaining accurate relapse data on drug abusers and understanding the factors influencing relapse. In summary, this study focuses on seeking to determine the accurate relapse rate of drug patients after compulsory detoxification, assessing the impact of aftercare services and locating other influencing factors on the relapse rate.

## Materials and Methods

### Sample Frame

In this study, drug patients released from Guangzhou T Compulsory Isolated Detoxification Center (hereinafter referred to as “T Center”) are taken as the overall sample frame. T Center has the largest number of drug abusers under compulsory detoxification in Guangdong Province and only serves male drug abusers. As of 2019, there were 32 centers for compulsory isolated detoxification in Guangdong, with a total of nearly 37,000 registered drug abusers, of which 28 centers admitted only males, 2 centers admitted only females, and 1 center admitted only underage drug patients. In addition, 1 Special Center of Compulsory Isolated Detoxification in Guangzhou specifically admits drug patients in Guangdong who have severe infectious diseases, have severe physical disabilities and are experiencing severe mental illness^2^ Before being admitted to T Center, all male drug patients undergo detailed physical examinations in a specialized hospital—Hospital of Guangzhou Drug Rehabilitation Bureau—to rule out those with severe infectious and mental diseases. The drug patients received by T Center are “ordinary” male drug abusers without serious health problems who have a certain degree of self-awareness and good self-care ability.

T Center was chosen for this study mainly for the following two reasons: ①It receives drug abusers in Guangdong Province and has the most drug patients in the province, so it can reflect the general situation of drug abusers. ②It is the first center in the province to provide drug patients with tracking services in the form of government purchases of social services. Due to the sensitivity of drug patients, it is difficult for ordinary researchers to contact them. T Center provided a feasible path for the operation of the tracking study by entrusting Guangzhou Shangshan Social Service Center to track and provide corresponding services to drug patients after release from T Center. Other centers of compulsory isolated detoxification had not yet tracked patients and provided them with services and therefore did not satisfy the requirements of the study. Notably, female drug abusers were not included in the study, mainly due to restrictions on administrative permission. At present, there are ~600 female drug patients in two women's compulsory rehabilitation centers in Guangzhou Province, but no plans or funds of these two centers supported tracking and aftercare, and the research team could not obtain research permission from these two centers. This is one of the main limitations of the study, and we suggest that further studies track female drug patients if situations permit in the future.

In 2016, T Center received a total of 602 male drug abusers, and currently, there are ~2,000 drug abusers under compulsory detoxification there. After the drug patients are admitted to the T Center, the professional medical staff enforce physical abstinence from drugs and treat and monitor their abstinence symptoms, physical health, and psychological and emotional changes. Drug abusers generally receive 1–2 years of drug addiction treatment at T Center, and 1 month after expiration of the compulsory detoxification period, those who pass the diagnostic evaluation are released from compulsory isolated detoxification.

### Relapse

In this study, relapse refers to the reuse of drugs by drug patients after compulsory detoxification, including taking drugs in secret without addiction and reusing drugs resulting in addiction. Since August 2016, the research team has established and signed a long-term research cooperation agreement with T Center, and T Center entrusts the research team with conducting diachronic follow-up visits with drug patients released from the center. The research team conducted follow-up visits with each drug patient released from compulsory detoxification after August 2016 at 1 week after release, 1 month after release, 3 months after release and 6 months after release (at time intervals of 3 months thereafter) to determine whether drug use had been resumed. The project team adopted four main methods to follow up regarding relapse: ① Self-narration by drug patients after compulsory detoxification: through interviews, home visits, telephone calls, WeChat, QQ, etc., the drug patients narrated their own situation with respect to drug resistance and answered interview questions^3^. ②Feedback interviews with family members or cohabitants on the relapse situation of drug patients. ③Verification of the drug patients' situation by anti-drug social workers and subdistrict offices: the project team took the initiative to contact the subdistrict offices and local anti-drug social workers^4^ in the places where the residences of drug patients were located to verify the truth of their self-narration and conduct multifaceted information verification. ④ Urine, hair or blood test reports provided by the public security organ: the research team contacted the public security organ through various channels and sought to obtain regular urine, hair or blood test reports for the drug patients; the research team also tried to encourage the drug patients to provide their drug test reports to verify whether relapse had actually occurred. Of the above four methods, the research team adopted at least two to confirm the relapse status of drug patients after compulsory detoxification to ensure the validity of the collected information.

### Questionnaire

To improve the follow-up rate and reduce the attrition of drug patients released from compulsory detoxification during the follow-up process, the research team entered T Center 1 month prior to the drug abstainers' release from compulsory detoxification, collected their personal and family contact details and addresses, and obtained their signed informed consent forms for this research project.

In addition, according to the purpose of the study, a structured questionnaire was adopted to collect the personal information and data of those to be released from compulsory detoxification, including the following four major aspects:

### Sociodemographic Information

Age, household registration (“1” = “within Guangzhou,” “2” = “within Guangdong Province (outside Guangzhou),” “3” = “in other provinces”), marital status (“0” = “married,” “1” = “single”), education level (“1” = “primary school and below,” “2” = “junior high school,” “3” = “senior high school and above”), relative economic status of the family (“0” = “affluent/normal,” “1” = “poor”), and employment before entering the compulsory isolated detoxification center (“0” = “employed,” “1” = “unemployed”).

### History of Drug Abuse

The age at first drug use, type of drug taken (“1” = “traditional,” “2” = “new,” “3” = “mixed”), whether this was the abstainer's first compulsory detoxification (“0” = “yes, it is the first time,” “1” = “no, compulsory detoxification undergone several times”) and whether the abstainer had previously been arrested for a crime (“0” = “no,” “1” = “yes”). Regarding the classification of drugs, the *China Drug Situation Report 2020* (3) divides drugs into two categories: synthetic drugs and opioid drugs. Synthetic drugs mainly include methamphetamine, K powder, ecstasy, triazolam, magu, etc.; opioid drugs include heroin, opium, morphine, marijuana, cocaine, etc. However, from the perspective of drug patients, drugs are more commonly divided into traditional drugs (heroin, morphine, marijuana, etc.) and new drugs (methamphetamine, K powder, ecstasy, etc.). In addition, some drug users will mix traditional drugs with new drugs (combining heroin and methamphetamine, magu and methamphetamine, etc.) to increase pleasure and experience stronger stimulation. Therefore, in this study, to facilitate the understanding of drug patients and based on the actual situation of drug use, the drugs used by drug patients are divided into three categories: traditional, new and mixed.

### Motivation for Drug Rehabilitation Prior to Release

The Stages of Change Readiness and Treatment Eagerness Scale (SOCRATES) (23, 24) was used to measure the willingness of those under compulsory detoxification to abstain from drugs. The SOCRATES was developed in the 1990s and was originally used to assess the motivation stages of alcohol addiction (24). The scale contains 19 items with a score of 1–5 and three measurement dimensions: recognition, ambivalence and taking steps. The total score on the scale is obtained by adding the scores on all the items. The higher the total score is, the stronger the motivation to abandon drugs is. The scale has good reliability and validity (25, 26) and is currently used in many countries (27, 28). The SOCRATES can also be used to assess the abstention motivation of addicts such as drug abusers (29) and people addicted to tobacco (30). The SOCRATES has been widely used in China and has good reliability and validity not only for alcoholics (31) but also for drug abusers (23). In the sample of this study, the internal consistency reliability of the 19 items was *Cronbach's alpha* = 0.830, which also indicates good reliability for drug patients under compulsory isolated detoxification.

### Clinical Situation

In addition to the questionnaire, this study obtained administrative permission from T Center to collect and record the drug patients' diagnosis results regarding physical health, anxiety and depression from the physicians before the patients were released from T Center. Physical health at the time of release from T Center was recorded as “0” = “healthy” and “1” = “general/unhealthy.” In addition, if the physical health assessment of a drug patient was “poor,” the corresponding specific disease information was also recorded. The diagnosis of anxiety and depression was performed by physicians using the *Hospital Anxiety and Depression Scale* (HADS). The HADS was compiled by Zigmond and Snaith (32) and is widely used in screening patients for anxiety and depression in general hospitals. Ye and Xu (33) translated it into Chinese and verified that it has good reliability and validity in China. At present, the HADS has been widely used in the assessment of anxiety and depression in patients with various clinical diseases (34). The HADS includes 14 items of 0–3 points, of which 7 items assess anxiety and 7 items assess depression. Subscale scores can be calculated separately or added together to calculate the total score on the scale; after converting some of the reverse test questions, the higher the total score is, the higher the patient's anxiety and depression are. In this study, the drug patients' scores on the anxiety and depression subscales assessed by the physicians before release were recorded separately.

### Ethical Considerations

Before the research started, the detailed research plan was submitted to the Academic Ethics Committee of the Public Administration School of Guangzhou University and the Education Section of T Center for review, and an ethical consent form was obtained. Informed consent forms and questionnaires were completed at T Center in a centralized manner by means of the real-name system. The researchers entered T Center on the first Tuesday of each calendar month and gathered all the drug patients who were to be released from the center within the same calendar month in the form of release education, and the patients filled in the questionnaire after completing the release education. First, the researchers explained in detail the purpose of this research and follow-up matters after release, emphasizing the principle of voluntariness and confidentiality. Drug patients could choose whether to participate in the study and whether to fill out the questionnaire. Second, all the drug patients agreeing to participate in this study needed to sign an informed consent form at the site, and the research team prepared an independent case file for each participant and archived the form. Third, in the process of information collection, the research team members answered participants' questions and gave guidance on site, and these forms and questionnaires were immediately collected upon their completion and were not handed over to the police at T Center. Finally, none of the data used in the analysis present or disclose any personal information of drug patients, and the data are only used for group analysis. All the data collected in this study are strictly confidential; if a third party outside the research team consults such data, any personal information that can identify drug users will be concealed.

### Experimental Design

The drug patients were randomly divided into two groups: the experimental group and the control group. The difference between the groups is that direct assistance was provided by social workers on the research team to those in the experimental group after release from T Center based on their difficulties in the course of follow-up, whereas only relapse follow-up was conducted for those in the control group, and no other intervention measures were taken.

The drug patients' placement in the experimental group or the control group was determined by adopting a completely random method when the questionnaires were collected. First, the numbers of drug patients in the experimental group and control group were determined based on the workload of the social workers on the research team. T Center releases 30–50 drug patients from compulsory detoxification each month, and it is estimated that 400–600 cases are tracked in a year. With 10 social workers on the research team, each social worker needed to follow up with ~50–60 drug patients (experimental group + control group) on average within 1 year and was responsible for professional services for ~20 drug patients (experimental group) at most. Therefore, the numbers of drug patients were ~200 in the experimental group and ~400 in the control group, and the ratio of the number of drug patients in the experimental group to the control group was determined to be 1:2. Second, when collecting questionnaires monthly, according to the order of the drug patients' seat numbers, the drug patients whose seat numbers were multiples of 3 were selected for the experimental group, and the rest of the drug patients were in the control group.

According to the literature, stable accommodations (16), effective financial assistance from the family (35) and psychological and emotional counseling (36, 37) can effectively reduce the occurrence of relapse among drug abstainers. Regarding the professional service follow-up provided to those in the experimental group, it was necessary to combine these services with available detoxification resources and the existing efforts of the subdistricts. However, due to the limited resources of the research team, it was not possible in this study to develop several service plans with proven effectiveness. For example, cognitive behavioral therapy can effectively reduce the relapse rate of those abstaining from opioid drugs (38). Therefore, the professional service intervention for the experimental group involved three main aspects: accommodation arrangement and application, temporary economic relief, and psychological and emotional counseling after the drug patients returned to the community. The social workers on the research team established contact with the subdistrict offices of the communities where those in the experimental group were located and assisted them in coping with life and solving the difficulties they faced through home visits, a telephone hotline service and other methods^5^ For those in the control group, due to the limited number of social workers on the research team and the limited research resources, several forms of follow-up were carried out according to the predetermined time points of the study, but no professional services were provided for them, and subsequent follow-up will be carried out when sufficient research resources become available. At present, the research team has a total of 10 full-time social workers, and each holds a social worker certificate and has completed drug rehabilitation service training.

### Sample Size

From September 2016 to September 2017, the total number of drug patients released from T Center was 510, and none refused to participate in the study. This might be due to the following two reasons: ① Due to the highly authoritative environment in the compulsory isolated detoxification center, drug patients are accustomed to obeying orders in the center. Although the principle of voluntariness was emphasized, they may still have considered participation in this research to be an order, especially with the presence of police officers nearby. ② Long-term isolation for drug rehabilitation isolates drug patients from normal society. Even if they return to the community after being released, they may suffer multiple types of discrimination due to their drug abuse background, and they need normal social communication and social care (39, 40). When introducing this study to drug patients, it was emphasized that social workers would care about their lives after they were released, which was conducive to drug patients' consideration of follow-up more positively and became an important source of their feelings of social care, thus enhancing their willingness to participate in this study. Judging from the actual follow-up situation in this study, the members of both the experimental and the control groups took a welcoming attitude toward regular follow-up contact with the social workers.

Ultimately, 153 drug patients were included in the experimental group, and 357 were included in the control group. Of the 510 drug patients released from compulsory detoxification, 24 were released before the research team collected the questionnaires, and the drug detoxification police at T Center collected their contact information and received signed informed consent forms from them, but no questionnaire data were obtained from them. During this time period, a total of 510 drug abstainers released from compulsory detoxification were tracked in this study. Because the questionnaire data were not obtained from the above 24 drug abstainers prior to their release from compulsory detoxification, they were only included to calculate the relapse rate but were excluded from the subsequent descriptive and statistical analyses, such as the analysis of survival time.

### Statistical Methods

First, this study conducts descriptive statistics on the sociodemographic characteristics, past drug abuse history, clinical information, and motivation for drug rehabilitation of the drug patients before they were released from T Center. It presents the overall group characteristics of the drug patients, performs a baseline assessment of the experimental group and the control group, and compares the differences in the characteristics of the two groups. Second, the overall relapse rate of the drug patients released from compulsory detoxification and the relapse rate of the experimental and control groups are reported based on the follow-up situation, and the frequency of relapse in each time period after their release is calculated.

Third, the Bayesian MCMC algorithm is used to estimate the average survival time of the drug patients before relapse, and data imputation is performed to obtain the specific survival time of each drug patient before relapse. The MCMC algorithm is commonly used in Bayesian survival analysis to extract posterior distribution random numbers or perform numerical simulations (41). It was proposed by Metroplis in 1953 (42). The algorithm first constructs a suitable Markov chain and then uses the Monte Carlo method for integral calculation to obtain the posterior distribution of the parameters (43). The specific process of the MCMC algorithm includes determining the prior distribution of parameter values, determining the value of the current parameter, using Gibbs sampling to obtain the value of the next parameter to be assessed according to the value of the currently assessed parameter, calculating the posterior probability, model iteration, convergence and other steps. In the setting of the prior distribution, this study adopts Murthy and Qi's suggestions on the prior distribution setting in the MCMC survival analysis (44, 45) and sets the prior distribution to the Weibull distribution, thus estimating the parameter value of the survival time to relapse with the MCMC method. This study uses the Bayesian MCMC method to estimate the survival time, mainly based on the following considerations: using the Bayesian MCMC method can obtain reliable estimation results through prior information and sample information and can simulate the posterior distribution of the parameters more conveniently (46). In addition, traditional survival analysis models have high requirements for data and require that there not be too many missing data because limited data may lead to low confidence levels or inaccurate estimates (47). Due to the particularity of the study, drug patients without relapse were considered censored data, so the MCMC method is more appropriate.

After survival analysis using the MCMC method, the Bayesian interpolation method is used to perform value imputation for the specific survival time of each drug patient 10 times to obtain the specific survival time of each drug patient. The basic idea of the Bayesian imputation method is to devise the interpolation model parameter from a random value of its posterior distribution (48). As in the previous MCMC survival analysis, the posterior distribution of the model is simulated (49), and this distribution can be used directly to impute the specific survival time to relapse of each drug patient when Bayesian imputation is used (49). Compared with the traditional OLS for imputation, the Bayesian imputation method can make full use of the limited data set for imputation, the data requirements are lower than those of the OLS method, and there are studies noting that the effect of the imputation of public health survey data is better than that of the OLS method (50). Kong et al. (48) compare the Bayesian imputation method with several other novel imputation methods, namely, the jackknife method and bootstrap method. The results of these three methods are relatively close after interpolation, but the confidence interval of the Bayesian interpolation method is narrower, which shows that the estimation accuracy of the Bayesian method is higher under the same confidence level.

Finally, for the specific survival time data obtained after imputation, the survival time is divided into quantiles according to the data distribution, and logistic regression is used to make statistical inferences on the factors affecting relapse. Due to the nonnormal distribution of the drug patients' survival times, the survival time range of all the drug patients is 104, with an average value of 220.35. The survival time difference between the experimental and control groups is large, and the survival time distribution is extremely nonnormal (see the descriptive statistics in the next section for details). If the survival time series were used as the dependent variable for OLS estimation, the model inference results would be excessively affected by outliers, and the results would be biased. Therefore, according to the suggestions of Zhao et al. (51), the survival time is discretized into two levels, low (recorded as “0”) and high (recorded as “1”), for logistic regression analysis to identify the significant factors that affect the survival time for relapse.

SPSS 22.0 is used to create a database and conduct descriptive statistical analysis. The MCMC estimation method and Bayesian imputation method are conducted via AMOS 24.0, and the logistic model estimation method is conducted using SPSS 22.0.

## Results

### Prevalence Information

The average age of the drug patients was 34 years old (see Table 1), with their ages mainly ranging from 27 to 41; the oldest was 58 years old, and the youngest was 19. Nearly 70% of the drug patients were single; singles who had never been married accounted for 53%, and divorced singles accounted for 16%. The proportion of the drug patients whose registered permanent residences were located in other provinces was the highest, accounting for 43%. Nearly two-thirds (61%) of the drug patients had received a junior high school education, and nearly 60% believed that their families were impoverished compared to other families. More than half of the drug patients were unemployed prior to their compulsory detoxification.

**Table 1:** Prevalence information of drug patients released from compulsory detoxification.

**Variables**	***Percentage/Mean (SD)^a^***			**χ^2^/*t***
	**Overall**	**Experimental group**	**Control group**	
	**(*N* = 486)**	**(*N* = 139)**	**(*N* = 347)**	
**Sociodemographic data**
Age	33.99(7.027)	34.79(8.039)	33.66(6.564)	1.602^n.s.^
**Marital status**
- Married	30.9%	36.0%	28.8%	2.380^n.s.^
- Single	69.1%	64.0%	71.2%	
**Household registration**
- Within Guangzhou	38.3%	42.4%	36.6%	1.562^n.s.^
- Within Guangdong Province (outside Guangzhou)	18.3%	18.0%	18.4%	
- In other provinces	43.4%	39.6%	45.0%	
**Education level**
- Primary school and below	26.1%	21.6%	28.0%	2.113^n.s.^
- Junior high school	61.3%	65.5%	59.7%	
- Senior high school and above	12.6%	12.9%	12.4%	
**Relative economic status of the family**
- Affluent/normal	40.9%	54.0%	35.7%	13.628^***^
- Poor	59.1%	46.0%	64.3%	
**Occupation prior to compulsory detoxification**
- Employed	44.7%	38.1%	47.3%	3.349^n.s.^
- Unemployed	55.3%	61.9%	52.7%	
**Drug abuse history**
Age at first drug use	23.02(6.002)	22.40(6.362)	23.26(5.843)	−1.428^n.s.^
**Whether in compulsory detoxification for the first time**
- Yes	40.9%	38.1%	42.1%	0.639^n.s.^
- No	59.1%	61.9%	57.9%	
**Type of drug taken**
- Traditional	41.2%	38.8%	42.1%	0.474^n.s.^
- New	47.5%	48.9%	47.0%	
- Mixed	11.3%	12.2%	11.0%	
**Previously arrested for a crime**
- No	30.0%	35.3%	28.0%	2.515^n.s.^
- Yes	70.0%	64.7%	72.0%	
**Clinical situation before release**
**Physical condition**
- Healthy	57.6%	54.0%	59.1%	1.066^n.s.^
- Unhealthy	42.4%	46.0%	40.9%	
Mental condition (HADS)	8.44(7.284)	8.24(6.963)	8.52(7.417)	−0.388^n.s.^
- Depression	5.14(4.162)	4.91(4.088)	5.23(4.194)	−0.768^n.s.^
- Anxiety	3.30(4.028)	3.33(3.596)	3.29(4.194)	−0.927^n.s.^
**Motivation for drug rehabilitation (SOCRATES)**	72.47(7.124)	72.17(5.890)	72.59(7.567)	−0.598^n.s.^

*n.s., nonsignificant*,

*** *p < 0.001*.

a *SD, standard deviation*.

According to their drug abuse histories, the participants used drugs for the first time at age 23 on average, most used drugs for the first time within the age range of 17 to 29, and 25% had started using drugs when they were underage. It was not the first time for 60% of the drug patients to undergo compulsory detoxification, and some had undergone it several times. The proportion of those who took new drugs was the highest, at nearly half (50%), whereas the proportion of those who took traditional drugs remained high (41.2%). Among the drug abusers taking new drugs, methamphetamine (65.2%) and ecstasy (21.3%) were the most frequently used; heroin (76.8%) was the most frequently used traditional drug. Seventy percent of the drug users had committed crimes due to drug use and been arrested previously, indicating a high correlation between drug use and criminal behavior.

Patient health was obtained from the diagnostic reports provided by the patients' doctor before release. Immediately prior to their release, 42% of the patients were diagnosed with less-than-ideal health. Poor physical condition was mainly caused by long-term chronic diseases, mainly hypertension, gastrointestinal disease, lumbar disc herniation, tuberculosis, hepatitis B and C, etc. The drug patients' overall average HADS score for mental health was 8.44, the average score on the depression subscale was 5.14, and the average score on the anxiety subscale was 3.30. On the whole, there was no severe depression or anxiety before the patients' release. With 8 points as the threshold (34, 52), 24.5% of the drug patients had positive depressive symptoms before release, and 14.6% had positive anxiety symptoms before release. The motivation for drug rehabilitation scale scores (SOCRATES) ranged from 19 to 95 points, the drug patients' average score was 72.47, and the patients' motivation for drug rehabilitation prior to their release was at a medium-high level.

Comparing the above information of the experimental and control groups of drug patients, Table 1 shows that the two groups were basically at the same baseline level, except that the family economic situation of the experimental group (54.0% affluent/normal) was relatively better than that of the control group (37.5% affluent/normal). There were no significant differences in sociodemographic information, drug abuse history, clinical diagnosis before release, or motivation for drug rehabilitation.

### Relapse Situation

The relapse status of the 510 drug patients released from compulsory detoxification was tracked. From September 2016 to September 2017, a total of 243 drug patients released from compulsory detoxification reused drugs, regardless of the length of time since their release, and the relapse ratio was 47.6% (see Table 2). The drug patients released from compulsory detoxification were interactively classified by group and relapse; the relapse rate of the experimental group was 26.8%, and the relapse rate of the control group was 56.6%. The difference between the two groups was significant (χ^2^ = 38.090, *p* < 0.001). The compliance of the experimental group was significantly better than that of the control group.

**Table 2:** Relapse rate (*N* = 510).

	**Compliance**		**Relapse**		**χ^2^**
	**Number**	**%**	**Number**	**%**	
Overall	267	52.4	243	47.6	
Experimental group	112	73.2	41	26.8	38.090^***^
Control group	155	43.4	202	56.6	

*** *p < 0.001*.

As seen in Table 3, of drug patients who reused drugs after their release, 74.1% reused drugs within 1 week after their release, which was the time period with the highest relapse rate; the percentage that relapsed from the 6th to the 9th months after release was also relatively high (8.6%). In the control group, 80.7% of those who started to reuse drugs did so within the 1st week after their release due to the lack of professional service follow-up, whereas only 41.5% of drug patients who reused drugs in the experimental group started within the 1st week after their release. There was a significant difference in the distribution of relapse time periods between the two groups (χ^2^ = 38.090, *p* < 0.001).

**Table 3:** Time period of relapse occurrence.

**Time period of relapse occurrence**	**Overall**		**Experimental group**		**Control group**		**χ^2^**
	**Freq.^a^**	**%**	**Freq**.	**%**	**Freq**.	**%**	
1 day < <7 days	180	74.1	17	41.5	163	80.7	28.014^***^
8 days < <30 days	15	6.2	6	14.6	9	4.5	
31 days < <90 days	18	7.4	7	17.1	11	5.4	
91 days < <180 days	21	8.6	8	19.5	13	6.4	
181 days < <270 days	7	2.9	2	4.9	5	2.5	
271 days < <360 days	2	0.8	1	2.4	1	0.5	
Total	243	100.0	41	100.0	202	100.0	

*** *p < 0.001*.

a *Freq., frequency*.

The follow-up took the date when the drug patients were released from compulsory detoxification as the starting date, and return visits were conducted at fixed time points. For drug patients who complied with the laws and regulations, September 30, 2017, is taken as the ending date, the survival time (days) is obtained by subtracting the starting date from the ending date, and right censoring is then conducted. For those who reused drugs, the follow-up was conducted at a fixed time point; because it is difficult to accurately determine the specific relapse date, interval censoring is conducted for the time intervals for the occurrence of relapse. The interval censoring value is calculated based on the period between the tracking time node when relapse occurred and the last tracking time node before relapse occurred. Thus, if a patient released from compulsory detoxification had not reused drugs when he was followed up 3 months after release but had reused drugs when he was followed up 6 months after release, the interval censoring value of the drug patient is between 91 and 180 days, recorded as “91 < <180”. The original survival time interval to relapse of all the drug patients after compulsory detoxification is estimated by Bayesian estimation and the Monte Carlo (MCMC) algorithm to calculate the average survival time to relapse. As motivation for drug rehabilitation has a significant effect on relapse, strong detoxification motivation can effectively delay the occurrence of relapse (53, 54). With detoxification motivation as the independent variable^6^ and the original survival time as the dependent variable, a regression model is constructed, and Bayesian estimation is then conducted. When the model reaches convergence, the regression coefficient *r* = 2.629 is obtained, and the average survival time to relapse is 220.35 days (see Table 4). The occurrence of relapse is clearly extended by 2.629 days for every 1-point increase in the detoxification motivation score. Based on the regression model of detoxification motivation and original survival time, the experimental group and the control group are further differentiated, the regression path coefficients of the two groups are set to be the same, and Bayesian estimation is conducted again. The average survival time to relapse of the experimental group was 393.32 days, the average survival time to relapse of the control group was 175.10 days, and the survival time to relapse of the experimental group was significantly greater than that of the control group.

**Table 4:** Estimation of mean survival time.

**Mean survival time (days)**	**Relapse of drug patients released from compulsory detoxification**							
	**Mean**	**S.E.^a^**	**S.D.^b^**	**C.S.^c^**	**95% Lower bound**	**95% Upper bound**	**Min**	**Max**
Total number (*N =* 486)	220.35	0.174	12.547	1.000	196.162	245.826	174.662	277.736
Experimental group (*N =* 139)	393.32	1.331	45.567	1.000	314.901	494.798	272.369	624.494
Control group (*N =* 347)	175.10	0.176	12.778	1.000	150.909	201.023	120.359	227.202

a *S.E., standard error*.

b *S.D., standard deviation*.

c *C.S., convergence statistic*.

### Influencing Factors

With the Bayesian imputation method, based on the regression model of detoxification motivation and original survival time, data imputation is conducted 10 times for the specific survival time to relapse of each released drug patient, and the 10 imputation values are combined and averaged to obtain the specific survival time to relapse of each released drug patient. Although the specific survival time is a continuous variable, the numerical difference is very large (the minimum is 2.6 days, the maximum is 596.6 days), and its distribution is nonnormal, showing serious positive skewness. A large number of samples are stacked on the left side. The specific survival time value obtained after imputation is not suitable for direct linear regression analysis and is not conducive to checking through the residual normal distribution test (55). This study uses the “Upper and Lower 27% Rule” to mathematically divide the specific survival time (56, 57) to maximize the discriminative power of the quantile group and obtain two groups of the 27% quantile and 73% quantile. The survival time to relapse of drug patients released from compulsory detoxification below percentile 27 is set as a low survival time, and the survival time above percentile 73 is set as high. Taking the survival time to relapse of the groups (0 = “high survival”; 1 = “low survival”) as the dependent variable, sociodemographic, drug abuse history, clinical situation and experimental grouping factors are introduced into the logistic regression model in batches as independent variables. As seen in Table 5, only the sociodemographic variable is placed in Model I, and the prediction accuracy of the model is 61.5%, which is 11.5% higher than the 50% prediction accuracy of the null model (χ^2^ = 22.330, *p* < 0.01). Marital status has a significant impact on the survival time of drug patients. Taking single status as a reference (single = 1), single drug patients are ~3 times more likely to have a low survival time than those with married status (*OR* = 3.043), and their relapse occurs within a shorter time. Compared with drug patients with household registration in other provinces, the risk of relapse of the drug patients with household registration in Guangdong Province (excluding Guangzhou City) is lower in a short period of relapse time (*OR* = 0.468), but there is no significant difference in survival time for relapse between the drug patients with household registration in Guangzhou and those with household registration in other provinces. The sociodemographic variable is retained in Model II, and the drug abuse history variable is added at the same time. Marital status and household registration still have a significant impact. Whether the drug patient was undergoing compulsory detoxification for the first time also has a significant impact on the survival time for relapse: the drug patients experiencing multiple compulsory detoxifications were nearly twice as likely to relapse within a short period of time after release as those undergoing compulsory detoxification for the first time (*OR* = 1.918), and the probability of low survival time was greater. After the drug abuse history variable is added, the model prediction accuracy rate is 65.3%, which is 3.8% higher than that of Model I, but the model improvement is not significant (Δχ^2^ = 10.205, Δd*f* = 5, *p* = 0.069 >0.050). The clinical situation variable is then added to Model III, and it reveals that the physical and mental health of the drug patients had no significant effect on their survival time, and the OR values of both are close to 1. Finally, the experimental grouping variable of whether social workers were involved in the follow-up is substituted into the model, and the prediction accuracy of Model IV increases to 70.6%. Compared with Model II, the explanatory power of the model is significantly improved (Δχ^2^ = 32.535, Δd*f* = 3, *p* < 0.001). When controlling for other variables, the control group was over 8 times more likely to have a low survival time than the experimental group (*OR* = 8.010). Thus, the social workers' follow-up with the experimental group members after release had a positive effect on prolonging their survival time and reducing their risk of relapse. When experimental grouping is controlled for, single status, household registration in other provinces and multiple compulsory detoxification experiences still increase the risk of early relapse and low survival time to relapse. With the increase in variables in the model, the influence of marital status on relapse decreases, but the influences of household registration and the number of compulsory detoxifications increase. Controlling for other variables, professional service follow-up has the greatest influence on the survival time to relapse (*OR* = 8.010), followed by marital status (*OR* = 2.909), previous compulsory detoxification experience (*OR* = 2.249) and location of household registration (*OR* = 1/0.642 = 1.557).

**Table 5:** Logistic regression of survival time.

**Variables**	***OR (S.E.)^a^***			
	**Model I**	**Model II**	**Model III**	**Model IV**
**Sociodemographic data**
Age	1.025 (0.020)	0.990 (0.024)	0.990 (0.024)	0.990 (0.026)
Marital status—Single^b^	3.043^***^(0.311)	3.442^***^(0.321)	3.488^***^(0.323)	2.909^**^(0.337)
**Household registration—Other provinces**
- Within Guangzhou	0.703 (0.304)	0.719 (0.312)	0.728 (0.313)	0.642^*^(0.338)
- Within Guangdong Province (outside Guangzhou)	0.468^**^(0.370)	0.453^*^(0.381)	0.443^**^(0.383)	0.337^**^(0.41)
**Education level—Senior high school and above**
- Primary school and below	1.679 (0.448)	1.623 (0.465)	1.622 (0.465)	1.582 (0.489)
- Junior high school	0.704 (0.398)	0.644 (0.415)	0.631 (0.417)	0.649 (0.440)
Relative economic status of the family—Poor	0.871 (0.271)	0.875 (0.28)	0.913 (0.291)	0.708 (0.317)
Occupation prior to compulsory detoxification—Unemployed	1.076 (0.266)	1.056 (0.279)	1.085 (0.283)	1.502 (0.311)
**Drug abuse history**
Age at first drug use		1.049 (0.025)	1.050 (0.026)	1.041 (0.028)
Whether in compulsory detoxification for the first time—No		1.918^*^(0.336)	1.999^*^(0.345)	2.249^*^(0.369)
**Type of drug taken—Mixed**
- Traditional		0.889 (0.480)	0.881 (0.483)	0.819 (0.507)
- New		0.606 (0.491)	0.603 (0.492)	0.659 (0.516)
Previously arrested for a crime—Yes		1.217 (0.299)	1.210 (0.301)	1.127 (0.323)
**Clinical situation before release**
Physical condition—Unhealthy			0.888 (0.291)	0.963 (0.310)
Mental condition (HADS)			0.994 (0.020)	0.985 (0.022)
**Group—Control group**				8.010^***^(0.420)
Constant	−1.227	−2.018	−2.052	−3.423
Chi-square	22.330^**^	32.535^**^	32.875^**^	63.386^***^
Nag R-square	0.109	0.156	0.157	0.287
−2Log Lik	340.879	330.674	330.334	299.823
Predicted %	61.5	65.3	64.1	70.6

* *p < 0.05*,

** *p < 0.01*,

*** *p < 0.001*.

a *OR, odds ratio, S.E., standard error*.

b *The “single” group was used as the reference group, and the same is true of the following variables. The bold values in the table mean the coefficients of odd ratio are significant*.

## Discussion

After 1 year of follow-up, the overall relapse rate of drug patients released from compulsory detoxification was 47.6%. The 1st week after release was the time period with the highest relapse rate, which is the same as the findings of Bradley's 1989 report: most of 78 hospitalized drug patients relapsed 1 week after hospital detoxification (58). Estimated by the Bayesian estimation and MCMC algorithm, the average survival time to relapse was 220 days. The detoxification motivation prior to release had a positive impact on the survival time to relapse of drug patients after compulsory detoxification. The time of relapse was extended by 2.629 days for every 1-point increase in the detoxification motivation score. The average survival time to relapse of drug patients in the experimental group, who received professional assistance from social workers, was 393.32 days, and it was 175.10 days in the control group. The professional assistance provided after their release from compulsory detoxification had a positive effect on delaying their relapse. The drug patients' clinical information before release, poor health, anxiety and depression did not have a significant impact on their survival time to relapse after their release. Newton et al. (14), through investigating the relapse factors of 73 methamphetamine-dependent individuals, found that “pain avoidance” is not an important factor influencing relapse, which is consistent with the conclusion of this study. When the group of drug patients was controlled for, marital status, previous compulsory detoxification experience and household registration location significantly predicted the length of their survival time to relapse, as those who were single, had a previous compulsory detoxification experience and whose households were registered in other provinces had a higher risk of early relapse.

Returning to the community after release, drug patients will face a free world that is completely different from the highly enforced and disciplined nature of isolated drug rehabilitation centers. The drastic changes in the living environment make it difficult to maintain the effectiveness of compulsory isolated detoxification, and a large number of drug patients relapse within a short period of time after release. In combination with the above research findings, prevention of relapse among drug patients and delay of their relapse can be based on the following factors.

### Carrying out Socialized Drug Prevention and Control Measures for Key Populations

At present, the resources invested in drug rehabilitation work are far from sufficient for the large group of drug patients in China (59). Because it is difficult to increase the resources for drug rehabilitation in a short period of time and because there is high heterogeneity among drug patients, it is particularly important to accurately classify and rank the relapse risk of drug patients and invest the limited drug rehabilitation resources in drug patients who have a higher risk of relapse (14) to prolong the effect of compulsory isolation for drug rehabilitation as much as possible. From the study, drug patients who were single, had had multiple compulsory detoxification experiences and had household registration outside the province were the key follow-up and support targets. First, single drug patients are especially worthy of attention. Single status means that drug patients are less socially restrained and receive less social support. It is not realistic to require every drug patient to maintain a marriage or get married, but it is helpful to improve their family support through family education and encouraging drug patients to cherish family relations (60). Especially after 1–2 years of compulsory isolation for drug rehabilitation, drug patients need more family trust, care and encouragement after being released from a drug rehabilitation center. Positive family relations play a very important role in making drug patients stay away from drugs and preventing their relapse (61). Second, strengthening community-based supervision for those with multiple compulsory detoxification experiences will effectively delay the occurrence of their relapse. On the one hand, the failure experience of “compulsory detoxification—relapse—compulsory detoxification again” makes drug patients fall into a negative behavior pattern; on the other hand, it also weakens the deterrence and authority of compulsory detoxification for drug rehabilitation (62). It is difficult for drug patients to break out of this cycle by relying on their own strength, especially after the end of compulsory detoxification, which involves the disappearance of strong supervision, and the sudden emergence of expansive free space makes drug patients relax their self-discipline and their vigilance against drugs. Drug patients who have experienced multiple compulsory detoxifications should not be simply ignored after release. In combination with this study, it was found that the average survival time for relapse was 220 days. Effective and intensive supervision at the community level is necessary for drug patients for at least half a year after they are released and is of great benefit to delaying relapse. Third, drug patients with household registration in other provinces are likely to relapse in a short time after their release, and their resettlement is urgently needed. After drug patients are released from isolated detoxification centers, the first problem they face is housing. According to China's *Regulations on Drug Rehabilitation* (63), in principle, drug patients need to return to the place where their registered permanent residences or fixed residences are located. For drug patients with household registration in the same province, the place of household registration is often the same as the place of residence, and any resettlement problem after their release can be resolved, and their basic living conditions in the community can be guaranteed by the local government. However, for drug patients with household registration in other provinces, it is difficult to maintain their original residences in Guangdong Province due to isolation for drug rehabilitation. After release, they are usually sent back to their registered permanent residences in other provinces. They are typically people who were originally migrant workers who came to Guangdong to work and earn a living. After experiencing compulsory detoxification and being sent back to their registered permanent residences, they face severe difficulties and will soon move around the country. At the time of release, many drug patients with household registration in other provinces desire to stay in their previous place of residence before they entered the drug rehabilitation center. However, due to the limited number of rehabilitation centers^7^, drug patients with household registration in other provinces are forcibly sent back to their registered permanent residences; as a result, they soon move around. It is more difficult for drug patients with household registration in other provinces to obtain proper resettlement after release, and the social control over them is also weak, which increases their possibility of relapse in a short period of time.

### Strengthening Professional Service Follow-up for Drug Patients Released From Compulsory Detoxification

Drug patients released from compulsory detoxification will face the temptation of drugs and need to be resocialized [66]. Professional assistance interventions will effectively extend the survival time to relapse and reduce the possibility of relapse. In this study, the follow-up and assistance provided by social workers to drug patients in the experimental group involved only three aspects—accommodation, temporary economic relief and psychological emotional counseling—and there were no extensive community-based treatment services. Nevertheless, the relapse rate of the experimental group (26.8%) was significantly lower than that of the control group (56.6%), which received no professional service follow-up, and the survival time to relapse of the experimental group (393.32 days) was more than twice that of the control group (175.10 days). Twice the effectiveness will be achieved with half of the detoxification effort if more specific professional services can be provided according to the needs of drug patients after compulsory detoxification, especially timely intervention within the 1st week after their release, assistance coping with chaotic and dangerous periods after their return to the community, and stabilization of their family life after their release.

### Drug Detoxification Work at Compulsory Isolated Detoxification Centers Should Go Beyond Physiological Detoxification

It is also necessary to strengthen drug rehabilitation education and improve the detoxification motivation of drug abstainers to consolidate centers' detoxification effectiveness even after release. In recent years, although the Chinese government has vigorously advocated community-based drug rehabilitation and hospital detoxification, compulsory isolated detoxification still constitutes a large proportion of all drug rehabilitation work. The relapse rate of 108 drug patients who had received detoxification treatment in hospitals in Spain was as high as 72.2% within 6 months (12). This study shows that if the length of time after the end of compulsory detoxification is not considered, the relapse rate of drug patients released from compulsory detoxification is 47.6%; if the specific survival time after Bayesian interpolation is used as the basis, the relapse rate of drug patients released from compulsory detoxification within 6 months is 45.7% (the survival time is fewer than 180 days). In comparison, China's measures of compulsory isolated detoxification still have outstanding drug rehabilitation effects and positive functions (64). In contrast to the previous emphasis on physiological detoxification at compulsory isolated detoxification centers, this study shows that the strength of detoxification motivation has a significant correlation with relapse among drug patients after their release. Drug patients should be guided to recognize the dangers of drugs, reflect on the causes of their drug abuse, strengthen their confidence in their ability to maintain detoxification, and establish new life goals (65). Based on this study, it is recommended that compulsory isolated detoxification centers introduce more social workers or other professionals to provide professional services such as drug rehabilitation education and detoxification motivation cultivation. For those soon to be released from compulsory detoxification, this early intervention by social workers will be very beneficial to the service follow-up provided after their return to the community and will reduce their rejection of professional services, thus improving the effectiveness of professional services, reducing the relapse rate and delaying the occurrence of relapse.

This study presents the relapse rate of Chinese drug patients under compulsory isolated detoxification through longitudinal tracking data for the first time and identifies the significant factors affecting relapse. It not only provides guidance for relevant research in the future but also prepares reference data for the comparison of the effectiveness of different drug rehabilitation models. However, the results are not easy to generalize, as the population studied is a group of males with low severity of medical, addictive and psychiatric illnesses. For female or special drug patients who are suffering from HIV, schizophrenia, physical disabilities, etc., more targeted research is required.

## Data Availability Statement

The raw data supporting the conclusions of this article will be made available by the authors, without undue reservation.

## Ethics Statement

The studies involving human participants were reviewed and approved by the Ethics Committee of School of Public Administration, Guangzhou University. The patients/participants provided their written informed consent to participate in this study.

## Author Contributions

NL: designed and conceptualized the study. ZL and YX: performed the experiments. NL and YX: analyzed the data. NL and ZL: wrote the manuscript. All authors contributed to the article and approved the submitted version.

## Funding

This work was supported by T Compulsory Isolated Detoxification Center under Grant [GX16513020] and Guangzhou Social Work Research Center under Grant [1201610595].

## Conflict of Interest

The authors declare that the research was conducted in the absence of any commercial or financial relationships that could be construed as a potential conflict of interest.

## Publisher's Note

All claims expressed in this article are solely those of the authors and do not necessarily represent those of their affiliated organizations, or those of the publisher, the editors and the reviewers. Any product that may be evaluated in this article, or claim that may be made by its manufacturer, is not guaranteed or endorsed by the publisher.

## Footnotes

^1^“Severe crackdown on crimes” is a Chinese judicial term, which is a shortened version of “severe crackdown on criminal activities in a serious and fast manner in accordance with the law”. Since the reform and opening-up, China has launched a number of “severe crackdowns on crimes”. The most recent “severe crackdown” started on January 23, 2018, when the Central Political and Legal Committee held a national teleconference on a national crackdown on gang crimes, and ended at the end of 2020.

^2^Please refer to the website of the Guangdong Provincial Bureau of Drug Rehabilitation for details: http://gdjdj.gd.gov.cn/gdjdj/common/db/lxfs/content/post_2120438.html.

^3^During the follow-up of the study, the research team followed the principle of confidentiality and kept confidential the conditions of drug patients released from compulsory detoxification who participated in the interviews. Moreover, if drug patients reused drugs after compulsory detoxification, the research team advised them to stay away from drugs and warned them of the risk of being subjected to a compulsory isolated detoxification decision by the local public security organ instead of reporting them to the police.

^4^At present, the Pearl River Delta cities in Guangdong Province, such as Guangzhou, Shenzhen, Foshan and Dongguan, which take the district-level administrative region as the unit, are vigorously promoting anti-drug social workers' participation in community-based drug detoxification and community-based rehabilitation through government procurement of services. Drug patients released from compulsory detoxification are beyond the scope of community-based detoxification in principle, and only 39 of the 510 drug patients who had completed compulsory detoxification in this study were ordered to receive community-based rehabilitation, but drug patients are still intensively monitored by the community, and their situation is checked by the subdistrict anti-drug office every 6–12 months after they return to the community.

^5^For details on the professional services carried out by the social workers, please refer to the official website of Guangzhou Shangshan Social Service Center: http://www.020sssg.org/.

^6^Twenty-four drug abstainers had already been released from compulsory detoxification when the questionnaire survey on detoxification motivation was conducted, so detoxification motivation data were not obtained from them, and the total sample size was 486 in the following Bayesian statistical analysis.

^7^This refers to drug rehabilitation centers. At present, there is only one drug rehabilitation center in Guangdong Province (Guangdong Sanshui Kangfuyuan) that can provide short-term accommodation and temporary resettlement for drug users who have no fixed residence in the province after the end of compulsory isolated detoxification.
